# Supplementary material for: Fungal succession on the decomposition of three plant species from a Brazilian mangrove
Source: Sci Rep. 2022 Aug 25;12:14547. doi: 10.1038/s41598-022-18667-x (PMC9411622; doi:10.1038/s41598-022-18667-x)
Supplement: Supplementary file 1 — Supplementary Information. [file 41598_2022_18667_MOESM1_ESM.docx]

**Fungal succession on the decomposition of three plant species from a Brazilian mangrove**

Marta A. Moitinho^1,2^; Josiane B. Chiaramonte^1,2^; Laura Bononi^1,2^; Thiago Gumiere^3^; Itamar S. Melo^1^ & Rodrigo G. Taketani^2,4^*

**1-Laboratory of Environmental Microbiology, Brazilian Agricultural. Research Corporation, EMBRAPA Environment, SP 340. Highway—Km 127.5, Jaguariúna, SP 13820-000, Brazil.**

**2-College of Agriculture Luiz de Queiroz, University of São Paulo, Pádua Dias Avenue, 11, Piracicaba, SP 13418-900, Brazil.**

**3- Institut National de la Recherche Scientifique, Centre Eau Terre Environnement. 490, rue de la Couronne, Quebec City, QC, G1K 9A9, Canada.**

**4-** **CETEM, Centre for Mineral Technology, MCTIC Ministry of Science, Technology, Innovation and Communication, Av. Pedro Calmon, 900, Cidade Universitária, Ilha do Fundão, Rio de Janeiro, Brazil, ZC 21941-908**

*** - Correspondance author: College of Agriculture Luiz de Queiroz, University of São Paulo, Pádua Dias Avenue, 11, Piracicaba, SP 13418-900, Brazil. rgtaketani@gmail.com**


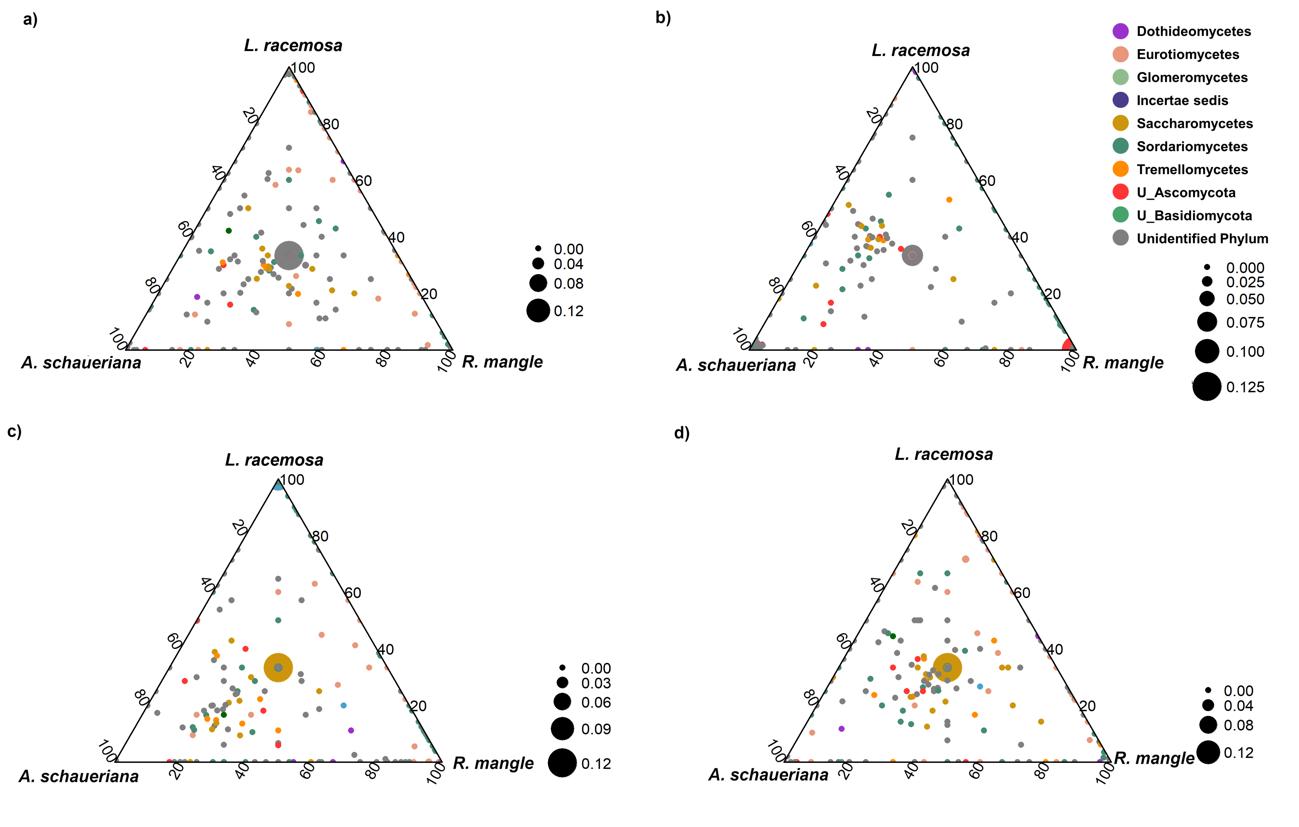


**Figure S1**: Ternary plots of the distribution of the OTUs across the three plant species (*A. schaueriana, L. racemosa* and *R. mangle*) along different times of the decomposition (**a)** time 7. **b)** time 15, **c)** time 30 and **d)** time 60). Each point represents an OTU, and its position indicates the proportion of its relative abundance at the different plant species. Points closer to the ternary plot corners indicate that a greater proportion of the total relative abundance of this OTU was found in this particular environment. Point colors indicate the phylum of the OTU. The lines inside the ternary plot indicate the X level of relative abundance of each of the sites. Only the OTUs with a per-site mean relative abundance of more than 0.1% are shown.

**Table S1**: Polimer composition of the leaves of mangrove trees from Cananéia, SP (Brazil)*.

|  | Hemicellulose | Cellulose | Lignin | Protein |
| --- | --- | --- | --- | --- |
| *A. schaueriana* | 147.46 ± 6.462 | 103.205 ± 14.333 | 221.095 ± 1.675 | 90.96 ± 0.028 |
| *L. racemosa* | 143.535 ± 8.746 | 100.425 ± 6.767 | 288.72 ± 123.220 | 85.22 ± 8.527 |
| *R. mangle* | 135.75 ± 19.756 | 101.38 ± 5.416 | 367.965 ± 11.151 | 90.625 ± 0.883 |

Data presented here correspond to the average followed by standard deviation of two replicates.
